# Low-carbon governance, fiscal decentralization, and enterprise green development: Evidence from China

**DOI:** 10.1371/journal.pone.0296490

**Published:** 2024-03-04

**Authors:** Shihai Liu, Jinsong Kuang, Dezhi Ding, Dag Øivind Madsen, Xiaofei Shi, Xianyang Fan

**Affiliations:** 1 Hunan University of Humanities, Science and Technology, Loudi, China; 2 School of Economics and Trade, Hunan University of Technology and Business, Changsha, China; 3 School of Economics and Finance, Shanghai International Studies University, Shanghai, China; 4 Davis School of Business, Colorado Mesa University, Grand Junction, Colorado, United States of Ameria; 5 Business Department, Hubei Branch, Bank of Communications, Wuhan, China; Hebei Agricultural University, CHINA

## Abstract

Simultaneously achieving economic development and environmental protection is a shared global challenge. While the positive effect of environmental regulations on protecting the environment has been widely recognized, the attention paid to low-carbon governance and corporate green transformation remains insufficient. Based on the two-stage least square regression model (2SLS) of instrumental variables, this paper utilizes panel data from China to identify the influence mechanism of government low-carbon governance on enterprise green development. It explores the effect of low-carbon governance on enterprise green development from the perspective of fiscal decentralization. The findings show that (1) Low-carbon governance significantly promotes corporate green development, primarily through improving industrial structure and technological innovation; (2) Low-carbon governance notably promotes the green development of private enterprises but has little effect on state-owned enterprises. There are also geographical differences, and the results are better in Eastern China than in the Central and Western parts of China; (3) Fiscal decentralization at both central and local levels inhibits the effect of low-carbon governance on driving corporate green development by causing a mismatch of human resources. Therefore, to promote corporate green development, low-carbon governance must prioritize green development, actively guide industrial structural upgrading and enterprise technological innovation, implement differentiated low-carbon governance measures tailored to different ownership enterprises, and optimize the assessment indicators for fiscal decentralization. This paper helps deepen the understanding of the relationship between government low-carbon governance and enterprise green development in developing countries. It can be used as a reference for government departments to formulate relevant policies.

## 1. Introduction

Environmental issues are among the world’s most important and pressing challenges today. Problems such as global warming, energy shortage, ecosystem degradation, and resource waste have caused significant damage to the earth and seriously threatened the sustainable development of human society [1, 2]. The Global Carbon Project, an international climate science body, released the "Global Carbon Budget 2022" report, which shows that China, the United States, India, and the European Union are the four economies with the highest carbon emissions from fossil fuels in 2022, with 11.4 billion tons, 5.1 billion tons, 2.9 billion tons and 2.8 billion tons, respectively. The rest of the world totaled 15.4 billion tons. The need to lower carbon emissions significantly impacts the ability to attain the Sustainable Development Goals. In fighting against carbon emissions, enterprises have become the main forces of carbon emission reduction and green innovation [3]. In addition, from a practical perspective, adopting green technology to achieve sustainable development is the basic social responsibility it should undertake and the basic path to shape new competitive advantages and open up markets [4].

In response to the rising pressure to reduce carbon emissions, the Chinese government has also adopted a positive response strategy, putting forward the major strategic goal of "striving to achieve the peak of carbon before 2030 and achieve carbon neutrality before 2060" to promote the green transformation and upgrading of enterprises. Low-carbon governance has played an important role in promoting the solution to the "carbon reduction dilemma" and achieving these green development goals. Low-carbon governance refers to a series of policies and actions implemented by local governments to reduce carbon emissions, including energy structure transformation, energy conservation and energy efficiency improvement, low-carbon transportation, carbon trading market construction, and ecological civilization construction. In the 21st century, China has implemented various low-carbon governance policies to reduce carbon emissions [5], and achieved remarkable results in promoting the green development of enterprises.

The low-carbon governance intensity of the government is a key factor affecting enterprises’ decision-making on carbon emissions. It has a crucial impact on the green development of enterprises. Regarding academic research, existing studies mainly discuss the impact of low-carbon policies on green development at the institutional level, such as carbon emission subsidies, carbon taxes, low-carbon pilot policies, carbon trading markets and technologies, etc [6–10]. There are also strands of literature examining the impact of low-carbon pilot policies on total factor productivity or green total factor productivity [11, 12]. However, at the government level, there are few studies on how low-carbon governance affects the internal mechanism of green development. When economic development is low, low-carbon governance policies may not reduce enterprises’ carbon emissions and promote green development. According to Porter’s hypothesis, as one of the important means of environmental regulation, low-carbon governance policies may have both a "compliance cost effect" and an "innovation compensation effect" on the technological innovation of enterprises, thus affecting the green development choice of enterprises.

In addition, facing the realistic background of central and local fiscal decentralization, the interests and needs of governments at all levels are inconsistent. If local government officials unilaterally pursue higher economic growth rates with the sole motivation of winning the political championship for official promotion, they will intentionally avoid low-carbon policies, and their behavior will be manifested as the government reducing the intensity of low-carbon governance [13–15]. This may harm the effect of implementing carbon emission reduction policies and the realization of green development of enterprises. In essence, local low-carbon governance determination and behavior are engines to promote ecological and environmental protection and green development of enterprises [8, 16]. Carbon emission reduction is a special public good. China’s low-carbon governance needs to focus on the government’s interests at all levels and the relationship between government and social governance. However, when people discuss the relationship between low-carbon governance and corporate green development, they tend to fail to consider the impact of fiscal decentralization.

Therefore, this paper adopts the two-stage least squares regression model (2SLS) of instrumental variables and the panel data of listed companies in China to identify the impact and mechanism of low-carbon governance on the green development of enterprises. Moreover, it explores the regulatory role of low-carbon governance in promoting enterprises’ green development from the fiscal decentralization perspective. As a result, this paper should be of great theoretical and practical significance in promoting carbon emission reduction strategy and realizing sustainable development goals both domestically and abroad.

Compared with the existing research literature, the contribution of this paper is as follows: First, combined with the evolution of China’s low-carbon governance policies, it recognizes the relationship between low-carbon governance and green development of enterprises, and the mechanism of action is further clarified, which provides theoretical reference and guidance on low-carbon governance to promote green development of enterprises. Second, it separates low-carbon governance from its multidimensional goals. A two-stage least square regression method based on instrumental variables was adopted (2SLS) since it not only alleviates the endogenous problem of reverse causation but also considers the means and effects of local government’s low-carbon governance and deepens the research on local government low-carbon governance. Third, considering that China’s low-carbon governance is characterized by top-down fiscal decentralization, the paper links low-carbon governance, fiscal decentralization, and enterprise green development and examines the moderate role of fiscal decentralization in the process of low-carbon governance to enterprise green development from the perspective of institutional environment change.

## 2. Literature review

In past research, scholars have explored the factors affecting green development from multiple perspectives, such as social responsibility, corporate governance, technological innovation, and green finance policy [17–20]. Regarding the research considering the impact of government environmental policies on corporate green development, the most well-known theory is Porter’s hypothesis [21, 22]. This theory explains the effect of environmental regulation on the green development of enterprises from two perspectives. In the short term, environmental regulation will increase the production cost and opportunity cost of enterprises, resulting in a "compliance cost effect." In the long term, environmental regulation will encourage enterprises to carry out technological innovation, leading to the "innovation compensation effect," shape the competitive advantage of enterprises in the market and help enterprises achieve sustainable growth and green development transformation. Furthermore, many scholars have also extensively explored the impact of carbon emission reduction policies on economic development [8, 16, 23]. Many of these results corroborate the two effects of "Porter’s hypothesis". For example, researchers have studied the impact of low-carbon city pilot policies on urban green total factor productivity.

The above-mentioned positive promotion effects are more pronounced in cities with higher urbanization and non-resource-based cities [12]. With the increase in carbon emission trading prices and the activity of the carbon emission trading market, carbon emission trading policy has played an increasingly important role in promoting the green development of manufacturing enterprises [24]. The carbon emission trading policy is for energy conservation and emission reduction. Still, excessive government intervention will lead to the failure of the carbon market mechanism, which will be detrimental to reducing carbon emissions from transportation [25]. The carbon emission reduction policy can promote the green development of enterprises, but the effect depends on the external environment. There may be the following two relationships between the government’s low-carbon governance and the green development of enterprises. On the one hand, the government’s low-carbon governance behavior increases the environmental protection pressure on enterprises through administrative, legal, and other means. To a certain extent, this will crowd out the funds enterprises use on green technology innovation, thereby inhibiting the green development of enterprises [26]. On the other hand, this environmental protection pressure can push enterprises to carry out green technology innovation and production mode transformation, increase the proportion of low-carbon production mode investments, and reduce the proportion of high-carbon production mode investments, shape the competitive advantage, and ultimately promote the green development of enterprises [27]. Taking research on low-carbon city policy pilots as an example, compared with nonpilot cities, low-carbon pilot cities will promote the development of a green economy through green technology changes [28].

In addition, according to the stakeholder theory, the relationship between the government and enterprises is similar to that between managers and stakeholders [29]. As a key stakeholder in ecological governance and green development, the government should place enterprises under the concept of green development in system design. Furthermore, the government should bring all stakeholders, including enterprises, into the top-level design of low-carbon governance, achieve the consistency of the government’s low-carbon governance objectives through coordination, coercion, or incentives, and finally achieve good governance effects [30]. The efforts of governments at all levels to implement low-carbon governance will be constrained by their interests, and lower governments do not always implement low-carbon governance according to the strict environmental policies of higher governments. Since the tax-sharing system was reformed in 1994, local governments have faced an institutional environment of economic decentralization and political centralization [31, 32]. To develop the local economy and government performance, fiscal decentralization will also impact the government’s low-carbon governance [33]. Its possible result is that government regulations seem to be getting stricter, and the efforts of low-carbon governance in different regions are not synchronized. This feature is fully verified by the heterogeneity of the impact of pilot cities on carbon emission reduction; that is, the policy effect is significant in Eastern China but not significant in Western and Central China. Compared with sub-provincial cities with higher administrative power, pilot policies in other cities have been more effective [34]. Furthermore, the low-carbon city pilot policy is conducive to the energy transition of southern, large, and resource-free cities [35]. Another example is that the improvement rate is relatively low in Beijing. At the same time, it is high in Tianjin, Hubei, and Chongqing, even though the carbon emission trading policy has significantly improved the green GDP of these cities [36].

To sum up, most of the existing studies in the literature discuss the impact of environmental regulations on green development, but few studies focus on in-depth exploration from the perspective of low-carbon governance. In addition, fiscal decentralization mainly refers to the source and form of fiscal revenue organized by the central government according to the items and scope of fiscal expenditure [37]. As an important part of the fiscal relationship between the central and local governments, fiscal decentralization may play an important role in the relationship between low-carbon governance and the green development of enterprises. Still, the research at this moment is relatively insufficient. Fiscal decentralization positively or negatively impacts green development in the short and long term [38, 39]. Previous studies have also confirmed that fiscal decentralization can significantly promote the growth of green total factor productivity, but this effect decreases with the increase of quantile value. Appropriate fiscal decentralization can improve green total factor productivity, while excessive fiscal decentralization will become an obstacle to the growth of green total factor productivity [40, 41]. However, there is scant research on the mechanism of fiscal decentralization regulation of low-carbon governance. Therefore, this paper begins with low-carbon governance and institutional environment, explores the relationship between low-carbon governance, fiscal decentralization, and enterprise green development, and tries to answer the following questions:

1. What is the effect of low-carbon governance on enterprise green development? What is the mechanism of action?
2. What is the moderating effect of the change of the institutional variable of fiscal decentralization on the above relationship?

A clear understanding of these problems is conducive to promoting the green development of enterprises and is conducive to the correct formulation of policies on the institutional environment by government departments.

The remainder of this paper is arranged as follows. The third part develops the institutional evolution and theoretical analysis. The fourth part constructs the measurement model and explains the data sources and variables. The fifth part reports the results of the regression analysis, the robustness test, the mechanism test, and the heterogeneity analysis. The sixth part is a further discussion after introducing fiscal decentralization. The seventh and last part of the paper provides a conclusion and identifies policy implications.

## 3. Institutional evolution and theoretical analysis

### 3.1 Institutional evolution of low-carbon governance

Since the beginning of the 21st century, China has implemented a series of low-carbon governance systems and policies at the national level to control carbon emissions. More than 20 related policies have been promulgated from 2003 to 2021 [5], such as China’s Action Plan for Sustainable Development (2003), Several Opinions on Promoting the Sustainable Development of Resource-Based Cities (2008), Carbon Emission Reduction Trading Management (2015), Energy-saving product certification Management Measures (2015), Carbon Trading Management Measures (2021), Opinions on Establishing and Improving the Value Realization Mechanism of Ecological Products (2021), Pollution Reduction and Carbon Reduction (2022) and so on. These systems and policies have been gradually improved, forming a policy support system led by binding targets, focusing on key industries and regions, including planning, law, administrative orders, pilots, market, fiscal and taxation, and other aspects. Starting from China’s 12th Five-Year Plan (2011–2015), the carbon emission intensity target was written into the Outline of the 12th Five-Year Plan for National Economic and Social Development of China, and China’s 13th Five-Year Plan (2016–2020) formed a multi-dimensional constraint target system including total energy, energy intensity, and carbon intensity.

In terms of policy types, it has gradually transitioned from administrative command-type to a situation where administrative command and market-type policies are equally important. It has initially explored the construction of investment and financing, and market mechanisms for addressing climate change and paid attention to the important role of pilot demonstrations in the policy formulation and implementation process. According to the analysis of the 13th Five-Year Plan for Ecological Environment Protection, during the 14th Five-Year Plan (2021–2025) period, China’s economic and social development is still unbalanced, uncoordinated, and unsustainable, with multi-stage, multi-domain, and multi-type ecological and environmental problems intertwined. Moreover, there is a large gap between the ecological environment and the people’s needs and expectations. Improving environmental quality, strengthening comprehensive ecological environment governance, and accelerating the improvement of ecological environment shortcomings are still the core tasks. We found that the Chinese government’s low-carbon governance policies include policies to reduce carbon dioxide emissions and policies to reduce other emissions. To sum up, the Chinese government’s low-carbon governance policy is a comprehensive environmental protection policy focusing on reducing carbon dioxide emissions, supplemented by reducing the exhaust emissions of enterprises.

### 3.2 Theoretical analysis

**Fig 1:**
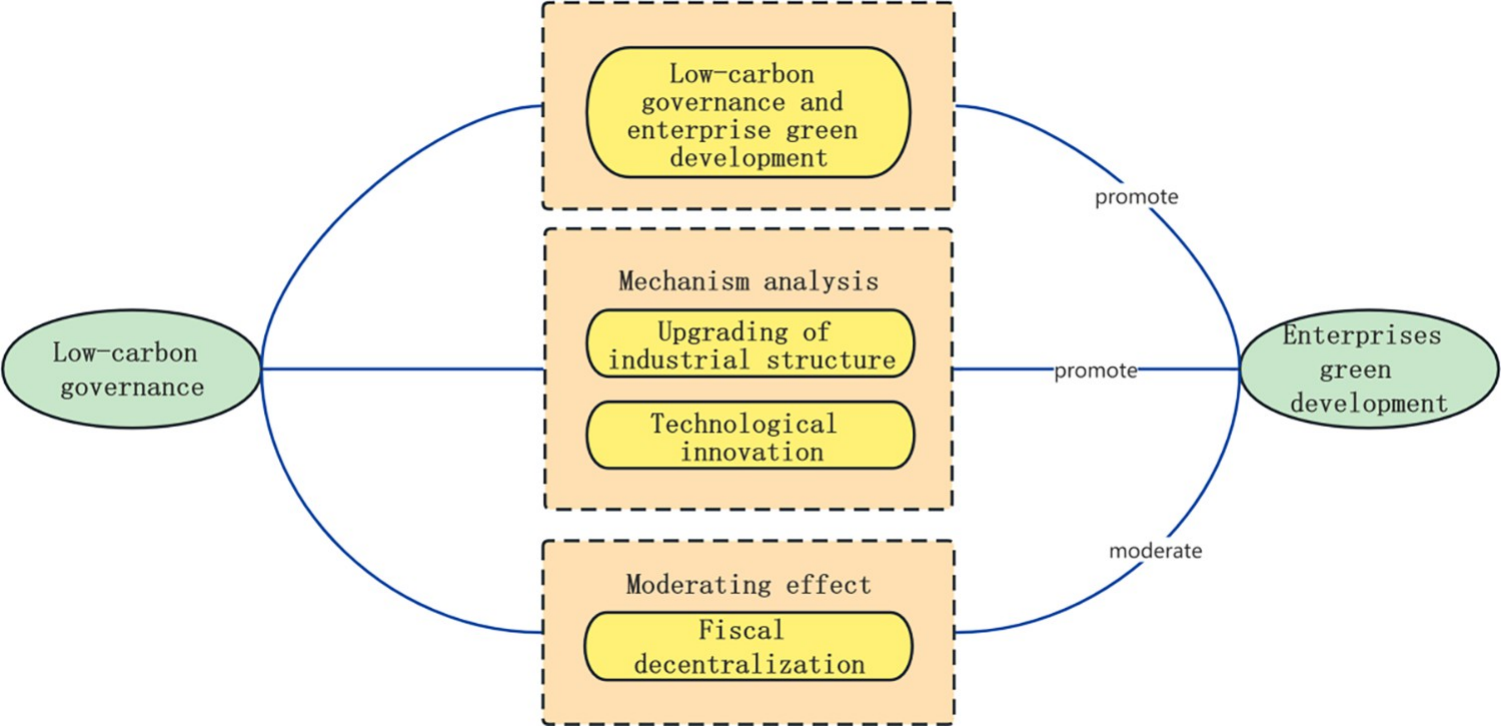
Theoretical analysis framework of low-carbon governance affecting the green development of enterprises.

This paper focuses on the impact of low-carbon governance on green development. In terms of theoretical research, it first analyzes the impact of low-carbon governance on the green development of enterprises. Then, it analyzes the influence mechanisms of low-carbon governance on the green development of enterprises from the two aspects of industrial structure upgrading and technological innovation. Finally, based on the institutional background of fiscal decentralization, it clarifies the moderating role of fiscal decentralization in the process of low-carbon governance driving the green development of enterprises. The theoretical analysis framework is shown in Fig 1.

#### 3.2.1 Low-carbon governance and green development of enterprises

As a policy tool to control carbon emissions, low-carbon governance promotes enterprises’ green transformation and upgrading through strict environmental policies and internalizing pollution costs [27]. Local governments can reduce total carbon dioxide emissions by taking command-controlled environmental actions such as ordering rectification, shutting down, or banning enterprises and projects that do not meet environmental standards [42]. Local governments could strengthen their enforcement of market-based environmental regulations by implementing pollution charges, environmental taxes, and emission rights trading. By doing so, they would increase the production costs of polluting enterprises, thereby raising the price of products and reducing the supply of products, thus achieving the goal of reducing total carbon dioxide emissions [43]. Since low-carbon governance affects the production costs and even the survival of enterprises, it also affects their production decisions. It forces enterprises to transform or improve the efficiency of the production process through technological innovation, which in turn significantly impacts the enterprises’ production and development modes [44]. Finally, the win-win goal of environmental quality improvement and economic green development will be realized [45]. Therefore, as an effective means of environmental protection, low-carbon governance can not only reduce carbon dioxide emissions to achieve the purpose of environmental protection but also encourage enterprise transformation and innovation through carbon emission trading and other measures, which will ultimately drive enterprises to green development [11]. Based on this, this paper proposes the following hypothesis:

*Hypothesis 1*: *Low-carbon governance helps promote the green development of enterprises*.

#### 3.2.2 Low-carbon governance, upgrading of industrial structure, and green development of enterprises

Low-carbon governance can optimize and upgrade the industrial structure mainly by influencing the entry and exit of enterprises and competition among enterprises [46, 47]. By setting up a negative list system for environmental access, the government defines the high-polluting industries prohibited from investing in and improves the quality of entering enterprises by setting environmental technical barriers. It also eliminates high-polluting and low-efficiency enterprises by shutting down and transferring them and guides potential or new entrants to invest in clean or high-tech industries. Through a series of adjustments, it can block the entry of some low-technology potential entrants or new entrants, making the production factor resources of high-pollution and low-efficiency enterprises return to the market, which then can supply high-efficiency enterprises, promoting the rapid development of high-quality enterprises and the upgrading of the industrial structure [48].

Industrial structure upgrading is a process in which production factors such as capital, labor, and land withdraw from low value-added, low efficiency, and high consumption production sectors or industrial chain links, and then import them into high value-added, high efficiency, and low consumption production sectors or industrial chain links [49]. The low-level industrial structure is often accompanied by the development mode of high input, high emission, high pollution, and low output, bringing more serious environmental pollution problems. This could seriously damage the ecological environment and is not conducive to green development [50]. Moreover, the high-level industrial structure represents a cleaner production mode, which can reduce pollution emissions and damage to the ecological environment and is thus conducive to green development [51]. At the same time, in the process of upgrading industrial structures, resources can be fully and effectively utilized. The unit input-output ratio can be improved, pollution emissions can be reduced, and sustainable and healthy economic growth can be promoted. Therefore, upgrading the industrial structure promotes the green growth of the economy [52, 53]. Accordingly, this paper proposes the following hypothesis:

*Hypothesis 2*: *Low-carbon governance helps enterprises develop green by improving the industrial structure*.

#### 3.2.3 Low-carbon governance, technological Innovation, and green development of enterprises

There have traditionally been two opposing views on the impact of low-carbon governance on technological innovation [54]. One view holds that low-carbon governance inhibits technological innovation, while the other holds that it promotes technological innovation. To be specific, the technological innovation of low-carbon governance is mainly reflected in that, in the face of the improvement of low-carbon governance, enterprises usually adopt such ways as purchasing environmental protection equipment or paying for environmental pollution costs to control environmental pollution. However, this will inevitably lead to additional human, material, and financial investment, and then squeeze out the investment of their research and development funds. Therefore, it generally weakens the ability of enterprises to carry out technological innovation. Meanwhile, when the external conditions of enterprises remain unchanged, the increase in production cost will lead to a decrease in profits, which in turn will further reduce the investment of R&D funds and is thus not conducive to the technological innovation of enterprises [55]. The promotion of technological innovation by low-carbon governance is mainly reflected in the following aspects. By increasing the implementation of low-carbon tax and other forms, low-carbon governance charges certain fees for the environmental resources used in enterprises’ production processes, internalizing the negative environmental externalities generated by enterprises and increasing their production costs.

Suppose enterprises want to win the initiative position in the fierce market competition. In that case, it is necessary to increase the support for technological innovation or introduce advanced technological equipment to improve enterprises’ technological level and the production process [56]. At the same time, in the context of the continuous improvement of low-carbon governance, the local government has given certain preferential fiscal and tax policies to enterprises engaged in technological innovation activities, thus reducing the cost of enterprises’ technological innovation research and development and improving the enthusiasm of enterprises for technological innovation research and development. Faced with the interaction of the two forces of increasing production costs and government innovation preferential policies, low-carbon governance can stimulate enterprises’ enthusiasm for technological innovation [57]. Technological innovation significantly promotes green development [58]. Specifically, through product and process innovation and technological progress, enterprises can improve the efficiency of resource use, reduce resource consumption and improve production efficiency, achieve the dual role of reducing environmental pollution and improving the technological content of products, improve the competitiveness of products, and thus meet the demands of consumers at a higher level, promote the increase of product output and drive the improvement of corporate profits, and achieving green development [59]. Based on this, this paper proposes the following hypothesis:

*Hypothesis 3*: *Low-carbon governance promotes technological innovation and green development of enterprises*.

#### 3.2.4. Fiscal decentralization, resource mismatch, and low-carbon governance

Fiscal decentralization enables local governments to have financial autonomy and receive the "residual claim" on fiscal revenues. Therefore, to compete for liquidity resources, local governments can implement public policies that suit their interests in a relatively independent manner [60]. Fiscal decentralization gives local governments the necessary resource allocation right to ensure the effectiveness of the incentive effect of the political promotion tournament.

In theory, local government competition stems from decentralization and has nothing to do with the regime, so it is not unique to China. As early as 1956, Tiebout discovered that as long as residents could move freely, the jurisdictional government must compete to attract "residents." This leads to the conclusion that "voting with enough" can bring hard constraints to the jurisdictional government [61]. Moreover, the "promotion tournament" for local officials, to compete for resources to achieve promotion targets has led to the persistence of "beggar-thy-neighbor" regional market segmentation [62]. To some extent, under the protection of local market segmentation, the market mechanism is broken, and outdated capacity has been maintained. Thus, it is difficult to exert comparative advantages and synergies between regions, which may exacerbate local CO2 emissions under the resulting resource mismatch and distortion, harming enterprises’ green development [63]. Among them, fiscal decentralization and market segmentation mainly lead to the mismatch of labor resources but have little impact on the mismatch of capital resources. Because capital resources, unlike labor resources, move in more diverse forms between provinces, they are harder for local governments to control. The low efficiency of labor resource allocation further becomes an obstacle for enterprises to achieve green development [64, 65]. In general, fiscal decentralization breeds market segmentation, which may cause the mismatch and distortion of labor resources. It means that fiscal decentralization and labor resource mismatch can affect the effect of government low-carbon governance. Accordingly, this paper proposes the following hypothesis:

*Hypothesis 4*: *Fiscal decentralization and mismatch of labor resources are not conducive to low-carbon governance and will inhibit the green development of enterprises*.

## 4. Econometric model and variable description

### 4.1 Model specification

The instrumental variable method, as one of the basic methods to solve endogenous problems, has been widely supported by the academic community or to alleviate the endogenous problems caused by the reverse causality of carbon dioxide emissions and green development [66]; this paper designed a least square regression model (IV-2SLS) based on instrumental variables by referring to Chen et al. (2018) and Deng et al. (2019) [67, 68]. The IV-2SLS method addresses endogeneity issues by utilizing exogenous variables as instrumental variables. The basic idea behind this method is to estimate the values of endogenous variables using instrumental variables in the first stage and substitute these estimated values into the original regression model, resulting in substituted data. Ordinary least squares (OLS) regression is performed in the second stage using the substituted data, yielding unbiased and consistent results. Referring to the IV-2SLS model, the basic operation idea of this paper is: (1) to build a two-stage measurement model; (2) In the one-stage regression model of instrumental variables (see Formula 1), the governance effect of low-carbon governance on carbon dioxide emissions was estimated; (3) In the two-stage instrumental variable regression, the carbon emission governance effect obtained in the first stage regression is taken as the core explanatory variable (see Formula 2) to investigate its impact on the green development transformation of enterprises. The economic implication is that if both are negative, it indicates that the government’s low-carbon governance has a positive effect on the green development of enterprises, otherwise, it has a negative effect. The specific model is as follows:

$${Carbon\_dioxide}_{it}=\alpha +\beta {FREQU}_{ct}+\theta \sum X_{it}+\varepsilon_{it}$$ (1)

$${GRE}_{it}=\alpha +\omega {Carbon\_dioxide}_{it}+\theta \sum X_{it}+\varepsilon_{it}$$ (2)

Where, Carbon_dioxide_it_ is the carbon dioxide emission of enterprise i in year t; FREQU_ct_ measures the low-carbon governance of region c in year t; GRE_it_ is the green development level of enterprise i in year t. X_it_ represents a series of control variables that may affect the green development of enterprises. E is a random disturbance term.

### 4.2 Selection of variables

(1) Enterprise green development (GRE_it_). This paper’s starting point is how to measure the green development of enterprises accurately. Most of the existing literature uses data envelopment analysis (DEA) to construct green total factor productivity to measure the green development of enterprises, but according to the connotation of industrial green development, enterprise green development should not only reflect the improvement of production efficiency but also reflect the reduction of environmental impact, resource utilization rate, carbon dioxide emission reduction, sustainable development and other indicators [69].

Therefore, to comprehensively grasp the connotation of enterprise green development, this paper takes the main indicators of industrial green development during the “13th Five-Year Plan” period in the “Industrial Green Development Plan (2016–2020)” issued by the Ministry of Industry and Information Technology in 2016 as the basis and constructs an indicator system that includes seven indicators: the data of comprehensive utilization rate of industrial solid waste, waste gas treatment level output value, water consumption per unit output value, sulfur dioxide emission per unit output value and industrial wastewater discharge per unit output value from the enterprise level [70, 71]. This paper comprehensively evaluates enterprises’ green development based on the entropy method.

(2) Low-carbon governance (FREQU_ct_). According to the research on the existing local low-carbon governance performance evaluation indicator system in, this paper, it is found that scholars have different emphases on the indicators they use to measure the government’s environmental governance level. Some scholars focus on pollution emission and treatment, mainly including industrial wastewater, waste gas, and solid waste emission and treatment; some scholars focus on the protection of greening and vegetation, mainly including per capita arable land area, forest coverage rate, per capita green space area, urban greening coverage rate, etc. Other scholars focus on the conservation of resources (or energy), mainly including energy consumption intensity, raw material consumption intensity, and water resource consumption intensity. It is our view that although these indicators can measure some aspects of the government’s environmental protection ability, they are more focused on reflecting the policy intensity, and it is difficult to reflect the government’s determination and strength of low-carbon governance accurately and comprehensively.

In the existing literature, there is no uniform standard for measuring environmental regulations, and the most commonly used indicators are mainly of three types: ① measuring by carbon dioxide emissions per unit output [72]; ② constructing an indicator system of environmental regulation according to different types of regulation such as administrative control, supervision, and economic regulation [73]; ③ measuring by pollution operation costs and investment costs per unit output [74]. Considering the research topic of this paper, the above environmental regulation indicators are highly related to industrial green development and have obvious endogeneity problems. In addition, since low-carbon governance has various forms, such as administrative orders and economic constraints, the above three types of indicators may be difficult to reflect comprehensive low-carbon governance. Therefore, this paper draws on the method of Chen et al. (2018) and uses the proportion of words related to “low-carbon” in local government work reports as a proxy variable for low-carbon governance [67]. On the one hand, it can reflect the intensity of the government’s low-carbon governance; on the other hand, it can alleviate the endogeneity problem because local government work reports generally occur at the beginning of the year, while economic activities run throughout the year, thus effectively avoiding the endogeneity problem caused by “reverse causality”. The reason is that under China’s institutional background, it is generally difficult for lower-level governments to directly influence higher-level governments’ decision-making [67]. This paper draws a kernel density plot of the frequency proportion of words related to “low-carbon,” and it can be found that the word frequency proportion increases year by year, indicating that local governments are constantly strengthening their policy determination and attention to the governance of carbon dioxide and other pollutants.

(3) Carbon dioxide emissions per unit output (Carbon_dioxide_it_). Carbon dioxide emissions per unit output are expressed by the proportion of carbon dioxide emissions to enterprises’ main business product sales revenue. The 2006 National Greenhouse Gas Inventory Guidelines prepared by the Intergovernmental Panel on Climate Change (IPCC) provide three methods for calculating energy consumption carbon emissions [75]. Due to the statistical data limitations of enterprise scale, most domestic and foreign energy consumption carbon emission studies are mainly based on the reference method based on apparent energy consumption [76]. The second volume of the IPCC National Greenhouse Gas Inventory Guidelines details the calculation formula for the reference method. Since carbon emissions from the energy sector are mainly due to the combustion of carbon-rich fossil fuels, carbon emissions can be determined by the amount of fuel and the emission factors of different fuels. For CO2 produced by combustion, combustion conditions (combustion efficiency, carbon residue in slag and ash, etc.) are relatively unimportant. Therefore, emission factors mainly depend on the carbon content of the fuel. Based on the above, this paper estimates carbon emissions from the energy consumption of enterprises according to the method of Chen et al. (2021) [77].

(4) Control variables (X_it_). Referring to the research by Yanwei Lyu et al. (2023), the control variables are mainly selected to reflect the enterprise and regional levels [20]. These authors mainly include ① enterprise-level variables. For example, enterprise size (lnsize). Existing literature shows that larger enterprises tend to make more stable environmental investments to ensure environmental compliance for sustainable development [78]. This paper uses the logarithm of total capital at the end of the year to measure enterprise size; enterprise age (age). Enterprise age usually represents the maturity of the enterprise, and existing studies show that more mature enterprises tend to have stronger management capabilities [79]. This paper uses the number of years of operation since the establishment of the enterprise to measure company age. Enterprise performance variables. Following Cai et al. (2019), Purbawangsa I B A (2020) and other studies, considering the impact of factors such as enterprise performance on enterprises, this paper also controls for enterprise capital intensity (capital), enterprise profit (profit), enterprise debt level (lndebts) and return on assets (ROA) [80, 81]. ② Regional-level variables. This paper refers to the practices of Tang C (2021), Peng X (2023), Zhang D (2021), and other scholars [82–84]. It selects variables such as logistics development level (LOGIS), telecommunications development level (COMMU), regional R&D level (RD), degree of openness (FDI), government expenditure level (GOV) and industrial structure (AIS). The above indicators are logarithmically processed to observe the trends and characteristics of the indicators easily.

### 4.3 Data sources

This paper integrates multiple sets of statistical data. Finally, it constructs a comprehensive database that includes data from the China Industrial Enterprise Database, the China Industrial Enterprise Pollution Database, and the provincial-level statistical data. The details are as follows.

One is the data from the China Industrial Enterprise Database. The data on China’s industrial enterprises come from the National Bureau of Statistics, covering all state-owned and above-scale industrial enterprises. This database contains basic information such as enterprise name, legal person code, enterprise address, and multiple financial indicators such as total assets and sales revenue. Following the practices of Brandt et al. (2012), Brandt et al. (2017), and others, the industrial enterprise database is processed as follows before matching: (1) excluding samples with duplicate legal person codes; (2) excluding samples that do not conform to the generally accepted accounting principles, such as current assets exceeding total assets, net fixed assets exceeding total assets, number of employees less than 10, etc.; (3) excluding samples with missing key indicators; (4) unifying the 4-digit industry codes from 1998 to 2014, which involved two industry classification adjustments in the above years, according to the industry correspondence table published by the National Bureau of Statistics unified in 2002; (5) constructing cross-year panel data by sequential matching method; (6) deleting samples with a large amount of missing data [85, 86].

The second source of data is from the China Industrial Enterprise Pollution Database. The China Environmental Statistics Database (CESD) is China’s most detailed environmental statistics data, covering about 85% of the main pollutant emissions yearly [87]. CESD contains enterprise basic information (such as enterprise name, legal person code, district code, and industry code), pollution emissions, coal and other petrochemical raw material consumption, and other environmental information of enterprises. For our empirical analysis, we use CESD information on coal and other petrochemical raw material consumption quantity, statistical year, ownership type, regional code, and industry code for analysis [88].

The third data source is the annual provincial-level statistical data produced by the National Bureau of Statistics of China, covering major socioeconomic statistical data of 30 provinces (Tibet is excluded from the sample due to severe data missing).

This paper uses economic data from Chinese enterprises and provincial levels from 2007 to 2014. It should be noted that since the data of the China Industrial Enterprise Database is only updated to 2014, our data interval also ends in 2014. Data on coal, gas, etc., come from the China Environmental Statistics Database (CESD), and other enterprise-level data come from the China Industrial Enterprise Database. Provincial-level data come from the annual statistical yearbooks of provinces. This paper uses legal person code, company name and company location to match China Environmental Statistics Database (CESD), China Industrial Enterprise Database (CEID) and prefecture-level city data by company location and year index. Further data screening and cleaning were conducted to improve the estimation’s effectiveness. Firstly, due to significant differences in the data, a logarithmic transformation was applied to the original data to mitigate the influence of dimension errors. Secondly, unreasonable data at the enterprise and regional levels, such as negative values for the number of employees in companies, were removed. Missing data were supplemented using interpolation methods. Thirdly, truncation was applied to all continuous variables at the 1st and 99th percentiles to mitigate the impact of outliers on the results. Descriptive statistics are displayed in Table 1.

**Table 1:** Descriptive statistics.

	(1)	(2)	(3)	(4)	(5)
VARIABLES	N	mean	sd	min	max
GRE	416,515	0.022	0.029	0.000	0.106
Carbon_dioxide	358,402	0.013	0.034	0.000	0.139
FREQU	416,515	0.183	0.052	0.098	0.286
scale_ass	416,174	11.241	1.515	8.785	14.232
age	416,316	2.292	0.617	1.099	3.555
capital	416,174	0.322	0.242	0.000	0.789
profit	295,779	2.227	0.219	1.757	2.557
lndebts	358,343	0.429	0.175	0.087	0.693
ROA	305,830	0.079	0.115	-0.064	0.397
logis	416,515	0.956	0.563	0.250	2.374
commu	416,515	2.419	0.045	2.274	2.476
fdi_zb	416,464	5.398	0.699	3.591	6.507
gov_zb	416,515	0.164	0.057	0.098	0.312
ais	416,515	0.444	0.041	0.352	0.508

## 5. Empirical results and analysis

### 5.1 Benchmark regression

This paper uses the instrumental variable method to estimate the impact of low-carbon governance on enterprise green development. In Table 2, models (1) and (2) report the baseline regression results. Among them, the estimated coefficients of low-carbon governance (FREQU_ct_) and carbon dioxide impact (Carbon_dioxide_it_) are both significantly negative, indicating that from a general perspective, low-carbon governance can reduce carbon dioxide emissions and significantly promote green development, which verifies hypothesis 1.

**Table 2:** Benchmark regression results.

	(1)	(2)
VARIABLES	GRE	Carbon_dioxide
Carbon_dioxide	-0.169***	
	(0.052)	
FREQU		-0.014***
		(0.001)
scale_ass	0.001***	0.001***
	(0.000)	(0.000)
age	0.001***	0.001***
	(0.000)	(0.000)
capital	0.007***	0.024***
	(0.001)	(0.000)
profit	-0.003***	-0.015***
	(0.001)	(0.001)
lndebts	0.004***	0.008***
	(0.000)	(0.000)
ROA	-0.001**	-0.010***
	(0.001)	(0.001)
logis	-0.001***	-0.001***
	(0.000)	(0.000)
commu	-0.001	0.016***
	(0.001)	(0.002)
fdi_zb	-0.001***	-0.003***
	(0.000)	(0.000)
gov_zb	0.006**	0.040***
	(0.002)	(0.002)
ais	0.002**	0.004**
	(0.001)	(0.002)
constant	0.022***	0.009**
	(0.002)	(0.004)
Observations	256,568	256,568
R-squared	0.698	0.146
Firm FE	YES	YES
Province FE	YES	YES
Year FE	YES	YES

Note: This table reports regression coefficients and z-statistics in parentheses for the full sample regression results. Continuous variables are winsorized at 1% and 99%. Firm/year fixed effects are included in the regression estimations. ***, **, and * represent a significance level of 1, 5, and 10%, respectively. The following tables all look like this one.

This paper draws on the method of Chen et al. (2018) [67]. It selects a low-carbon governance indicator (FREQU_ct_) with strong homogeneity as an instrumental variable to test the low-carbon governance, carbon dioxide emissions, and green development in the 2SLS framework. The results show that the Kleibergen-Paap rk LM statistic value is 6041.206, and the p-value is less than 0.01, which indicates that the null hypothesis of “insufficient identification of instrumental variables” is significantly rejected at the 1% level; the Kleibergen-Paap rk Wald F statistic value is 6399.485, which indicates that there is a high correlation between the endogenous variable and the instrumental variable, and there is no weak instrumental variable problem.

Many scholars have confirmed the correlation between low-carbon policy and enterprise carbon emissions [89–91]. The reason why the low-carbon governance variable used in this paper can better satisfy the exogeneity assumption of the instrumental variable is as follows: First, local government work reports generally occur at the beginning of the year, while economic activities run throughout the year, thus effectively avoiding the endogeneity problem caused by “reverse causality”; Second, the low-carbon governance variable in this paper is a provincial-level variable, while other related variables are enterprise-level variables, which helps to alleviate the endogeneity problem. The reason is that under China’s institutional background, it is generally difficult for enterprises to directly influence the decision-making of higher-level governments.

### 5.2 Robustness tests

#### 5.2.1 Eliminating the impact of municipalities

Since the four municipalities are directly governed by the central government and have a special administrative status, to eliminate the interference of administrative factors on the baseline regression results, this paper excludes Beijing, Shanghai, Tianjin, and Chongqing from the full sample and then regresses. The results are shown in columns (1) and (2) of Table 3, and the baseline regression results are still robust.

**Table 3:** Robustness test I.

	(1)	(2)
VARIABLES	GRE	Carbon_dioxide
Carbon_dioxide	-0.155***	
	(0.051)	
FREQU		-0.015***
		(0.001)
Constant	0.042***	0.106***
	(0.006)	(0.007)
Observations	238,671	238,671
R-squared	0.705	0.153
Firm FE	YES	YES
Province FE	YES	YES
Year FE	YES	YES

#### 5.2.2 Control potential missing variables

In terms of control variables, although we have controlled for the fixed effects of enterprises and years and the main indicators at the enterprise and regional levels, there may still be a problem with omitted variables. Therefore, we add enterprise-level variables, such as wage level (payable wage) and enterprise tax burden level (tax income), to the basic econometric model to further test the robustness. Heyman (2005) believes a significant positive correlation exists between wage-level differences and enterprise profits [92]. The overall level and hierarchy of employee wages will cause changes in enterprise performance, which may, in turn, have a corresponding impact on enterprise green development. This paper uses the logarithm of the total amount of wages payable by enterprises in the current year to measure the wage level of enterprises. Income tax payable refers to the tax that enterprises should pay from their income from production and operation and other activities according to the relevant tax laws of the state. Dang D (2019) believes that economic policy uncertainty is positively correlated with enterprise tax burden level, and the higher the tax quota, the stronger the impact [93]. Considering that the relevant government environmental governance policies may affect enterprises’ green development level through taxation, this paper uses the logarithm of income tax payable by enterprises in the current year to measure the tax burden level of enterprises. Therefore, to reduce the impact of these two potential omitted variables, this paper controls them. The results are shown in columns (1)-(2) of Table 4. After controlling for more enterprise-level variables, the regression coefficients are similar to those in the baseline regression table, indicating that potentially omitted variables do not impact the basic conclusions.

**Table 4:** Robustness test II.

	(1)	(2)
VARIABLES	GRE	Carbon_dioxide
Carbon_dioxide	-0.143***	
	(0.032)	
FREQU		-0.029***
		(0.001)
scale_ass	0.001***	0.001***
	(0.000)	(0.000)
age	0.001***	0.001***
	(0.000)	(0.000)
capital	0.006***	0.019***
	(0.001)	(0.000)
profit	-0.001	-0.009***
	(0.001)	(0.001)
lndebts	0.003***	0.007***
	(0.000)	(0.000)
ROA	-0.001	-0.007***
	(0.001)	(0.001)
logis	-0.002***	-0.001***
	(0.000)	(0.000)
commu	-0.005***	0.014***
	(0.001)	(0.002)
fdi_zb	-0.001***	-0.002***
	(0.000)	(0.000)
gov_zb	0.000	0.012***
	(0.001)	(0.002)
ais	0.001	-0.008***
	(0.001)	(0.002)
payable_wage	0.000	-0.001***
	(0.000)	(0.000)
tax_income	-0.000***	-0.000***
	(0.000)	(0.000)
Constant	0.029***	0.013***
	(0.003)	(0.004)
Observations	173,129	173,129
R-squared	0.736	0.172
Firm FE	YES	YES
Province FE	YES	YES
Year FE	YES	YES

### 5.3 Heterogeneity test

#### 5.3.1 Regional heterogeneity

Considering the large differences in economic development conditions and industrial bases among different regions, low-carbon governance may have heterogeneous effects on carbon emissions and the green development of enterprises in different regions. Here, we divide the sample into eastern, central, and western regions. The results in columns (1)-(3) of Table 5, show that the coefficients of the core explanatory variables in the eastern region are still significantly negative, indicating that low-carbon governance in the eastern region can reduce carbon dioxide emissions and thus significantly promote the green development of local enterprises. Low-carbon governance in the central region can increase enterprise carbon emissions but has no significant impact on enterprise green development, which is consistent with Zhang et al. (2021) and Wang et al. (2022) [94, 95]. The possible reason is that the central region has a low level of scientific research and openness, and enterprises find it difficult to achieve green development by reducing carbon emissions when facing low-carbon governance. Low-carbon governance in the Western Region inhibits enterprise green development. The possible reason is that the western region is economically backward, and the government prioritizes growth over environmental protection, so the governance effect has not achieved the expected purpose.

**Table 5:** Regional heterogeneity.

	(1)		(2)		(3)	
	Eastern Region		Central Region		Western Region	
VARIABLES	GRE	Carbon_dioxide	GRE	Carbon_dioxide	GRE	Carbon_dioxide
Carbon_dioxide	-0.333***		0.033		-0.270***	
	(0.059)		(0.078)		(0.094)	
FREQU		-0.019***		-0.021***		0.028***
		(0.002)		(0.004)		(0.006)
Constant	0.013***	0.006	0.114***	0.009	0.027***	0.014
	(0.003)	(0.005)	(0.015)	(0.036)	(0.008)	(0.017)
Firm FE	YES	YES	YES	YES	YES	YES
Province FE	YES	YES	YES	YES	YES	YES
Year FE	YES	YES	YES	YES	YES	YES
Observations	179,480	179,480	42,930	42,930	34,158	34,158
R-squared	0.596	0.132	0.764	0.146	0.602	0.183

#### 5.3.2 Heterogeneity of enterprise ownership

The ownership attribute of enterprises usually has different effects on their environmental strategies. Therefore, based on the baseline model, this paper divides the whole sample into three sub-samples: state-owned enterprises, private enterprises, and foreign-funded enterprises, and further examines whether low-carbon governance will have heterogeneous green development effects on different types of enterprise subjects.

The estimation results are shown in Table 6. Column (1) shows that low-carbon governance can reduce carbon emissions for state-owned enterprises but has no significant impact on green development. Column (2) shows that the estimated coefficients of low-carbon governance (FREQU_ct_) and carbon dioxide impact (Carbon_dioxide_it_) are both significantly negative for private enterprises, indicating that low-carbon governance can significantly promote green development for private enterprises. In addition, the estimated coefficient is not significant in column 3 for foreign-funded enterprises. This indicates heterogeneity in the green development effect of low-carbon governance at the level of enterprise ownership attributes. Low-carbon governance can more significantly reduce carbon dioxide emissions for state-owned and private enterprises, but private enterprises achieve this through green development, while state-owned enterprises do not. A possible reason is that state-owned enterprises have stronger path dependence effects and weaker R&D motivation. This is consistent with the research conclusions of Ren et al. (2019), Han and Sang (2018), and others. Ren et al. (2019) found that the emission trading system has a greater effect on improving the total factor productivity of non-state-owned enterprises than state-owned enterprises [96]. Han and Sang (2018) found that state-owned enterprises have less enthusiasm to switch their products to cleaner ones when facing environmental regulation constraints [97]. For foreign-funded enterprises, the effect of low-carbon governance is not significant, possibly because foreign companies have better environmental performance than domestic ones. Foreign-funded enterprises have relatively advanced technology, stronger management capabilities, and stronger environmental awareness, so they are not significant.

**Table 6:** Ownership heterogeneity.

	(1)		(2)		(3)	
	State-owned enterprise		Private enterprise		Foreign-funded enterprise	
VARIABLES	GRE	Carbon_dioxide	GRE	Carbon_dioxide	GRE	Carbon_dioxide
Carbon_dioxide	0.046		-0.276***		1.120*	
	(0.112)		(0.060)		(0.643)	
FREQU		-0.023***		-0.016***		-0.005*
		(0.008)		(0.002)		(0.003)
Constant	-0.005	0.046**	0.038***	0.020***	0.010	-0.015**
	(0.008)	(0.018)	(0.003)	(0.005)	(0.012)	(0.006)
Firm FE	YES	YES	YES	YES	YES	YES
Province FE	YES	YES	YES	YES	YES	YES
Year FE	YES	YES	YES	YES	YES	YES
R-squared	0.769	0.151	0.629	0.158	0.175	0.079
Observations	14,761	14,761	188,476	188,476	53,219	53,219

#### 5.3.3 Labor intensity heterogeneity

Labor intensity can reflect both the sensitivity of enterprises to labor and the dependence on technology, equipment, and resources, which in turn directly affect the green innovation development of enterprises [98]. Song J (2022) and Wang M (2021) both found that different degrees of labor intensity have a moderating effect on green development [99, 100]. For enterprises with different levels of labor intensity, the effect of low-carbon governance on enterprise carbon emissions and green development may differ. Therefore, this paper divides the sample into groups based on the mean value of labor intensity. The results in columns (1)-(2) of Table 7 show that low-carbon governance has a poor effect on carbon emission reduction for enterprises with high labor intensity and inhibits green development. The possible reason is that capital deepening is insufficient in enterprises with high labor intensity, there are large financing constraints, technology upgrading faces bottlenecks, and it is difficult to break through the dilemma of green transformation and upgrading. From the results in columns (3)-(4) of Table 7, it can be seen that low-carbon governance has a better effect on carbon emission reduction for enterprises with low labor intensity and promotes green development. The possible reason is that enterprises with low labor intensity have stronger R&D investment, green technology innovation effects are revealed, and thus the level of green development is improved. This result is consistent with Wang M (2021)’s research conclusion [100].

**Table 7:** Labor intensity heterogeneity.

	(1)	(2)	(3)	(4)
	High labor intensity		Low labor intensity	
VARIABLES	GRE	Carbon_dioxide	GRE	Carbon_dioxide
Carbon_dioxide	-0.2147***		-0.142***	
	(-3.22)		(0.034)	
FREQU		0.0194***		-0.025***
		(10.12)		(0.002)
Constant	0.0156***	0.0355***	0.025***	0.001
	(3.53)	(6.24)	(0.002)	(0.005)
Observations	67,573	67,573	189,030	189,030
R-squared	0.766	0.128	0.688	0.158

### 5.4 Mechanism test

This paper follows the method of Mao (2019) and constructs the following new three-stage least squares (3SLS) model to test the “structural effect” and “technological effect” of low-carbon governance [101]:

$$M_{it}=\alpha +\omega {Carbon\_dioxide}_{it}+\theta \sum X_{it}+\varepsilon_{it}$$ (3)

$${GRE}_{it}=\alpha +\rho M_{it}+\theta \sum X_{it}+\varepsilon_{it}$$ (4)

Where, *M*_*it*_ respectively represent the two mediating indicators of industrial structure (*HP*_*INDUS*_*it*_) and technological innovation (*CRSTE*_*it*_). Industrial structure (*HP*_*INDUS*_*it*_) is measured by the proportion of six major high-energy-consuming industries in industrial added value; technological innovation (*CRSTE*_*it*_) is measured by innovation efficiency calculated by input-output method, specifically, using industrial added value as the expected output, capital stock and employment as main inputs, and using entropy method to estimate [102]; the interpretation of other variables is the same as the baseline model, and the relevant data are from China Statistical Yearbook, China Industrial Enterprise Database, and China Industrial Enterprise Pollution Database. Table 8 reports the test results.

**Table 8:** Mechanistic testing.

	(1)	(2)
	*HP*_*INDUS*_*it*_	*CRSTE*_*it*_
Carbon_dioxide	0.051***	-1.345***
	(0.004)	(0.045)
logis	0.040***	-0.055***
	(0.000)	(0.006)
commu	-0.249***	0.771***
	(0.013)	(0.154)
fdi_zb	-0.023***	0.024***
	(0.000)	(0.005)
gov_zb	-0.019**	0.732***
	(0.009)	(0.107)
ais	-0.587***	1.623***
	(0.005)	(0.056)
age	-0.001**	0.027***
	(0.000)	(0.005)
capital	0.001	-1.134***
	(0.001)	(0.007)
profit	0.000	0.585***
	(0.001)	(0.012)
scale_ass	-0.003***	-0.034***
	(0.000)	(0.003)
lndebts	0.000	0.045***
	(0.001)	(0.010)
ROA	0.007***	1.315***
	(0.001)	(0.018)
_cons	1.343***	-3.268***
	(0.032)	(0.375)
N	256568	251625
r2	0.263	0.263

As shown in model (1) of Table 8, the estimated coefficient of carbon dioxide emission (Carbon_dioxide_it_) is significantly positive, indicating that government governance reduces enterprise carbon dioxide emissions by significantly reducing the proportion of high-energy-consuming industries, and thus promotes the green development of local enterprises, that is, it plays a significant “structural” effect of low-carbon governance. As shown in model (2) of Table 8, the estimated coefficient of technological innovation (*CRSTE*_*it*_) is significantly negative, indicating that government governance plays a significant “technological effect” by enhancing technological innovation to promote enterprise carbon dioxide reduction and green development. This result verifies hypotheses 2 and 3.

## 6. Further discussion: The effects of fiscal decentralization on low-carbon governance

This paper revolves around the theme of the governance effectiveness of low-carbon management on the green development of enterprises. From the analysis in the preceding sections, a roughly clear evolutionary path can be observed. On the one hand, governmental low-carbon governance can effectively facilitate structural improvements and technological advancements to promote the green development of enterprises. However, on the other hand, certain external factors may also play a significant role during this process, among which fiscal decentralization is particularly crucial [39, 41]. These studies show that the balanced process of "governmental low-carbon governance—enterprise green development" is continuously intertwined with the influence of fiscal decentralization. A lower degree of fiscal decentralization and a reduced labor resource mismatch promote enterprises’ green development. This section delves further into the perspective of fiscal decentralization, aiming to reveal its impact on the effectiveness of low-carbon governance in driving green development in enterprises.

Based on the incentive of "political championship" and the "short-sighted" behavior of economic development, there are significant variations in the intensity of low-carbon governance implemented in different regions. In competing for economic resources and implementing low-carbon governance, local governments engage in competitive behaviors that can lead to "beggar-thy-neighbor" actions [103], resulting in the misallocation of local resources due to competitive game equilibrium. To explore the effects of fiscal decentralization and resource misallocation, this study adopts a methodology similar to Li et al. (2018) ’s approach by incorporating the interaction terms of fiscal decentralization, resource misallocation, and low-carbon governance into Model (1) [91]. This, together with Model (2), constitutes a new 2SLS (Two-Stage Least Squares) model to examine the aforementioned mechanism. The specific model construction is as follows:

$$C{arbon\_dioxide}_{it}=\alpha +\beta FREQU_{it}+\mu FREQU_{ct}\times MIS_{it}\times FIN_{it}+\eta FIN_{it}+\eta MIS_{it}+\theta \sum X_{it}+\varepsilon_{it}$$ (5)

Where FIN measures the degree of fiscal decentralization in the region, fiscal decentralization refers to the autonomy of local governments to decide their own budget revenues, expenditures, and tax revenues, and to issue and manage government debt. This paper mainly uses the fiscal expenditure indicator to measure the degree of fiscal decentralization, because the level of fiscal expenditure decentralization reflects the financial power and expenditure responsibilities that local governments should have and bear, which is more consistent with the reality of China. Considering the impact of population size, this paper follows Xue Chengkai (2022) and calculates the per capita value of fiscal expenditure, using “the ratio of local per capita fiscal expenditure to central per capita fiscal expenditure” to measure the degree of fiscal decentralization [104]. Fiscal data are from the sum of the general public budget and the government fund budget. *MIS*_*it*_ respectively represent the capital mismatch index (*TK*_*it*_) and labor mismatch index (*TL*_*it*_), which are calculated according to Chen Yongwei and Hu Weimin (2011), and the interpretation of other variables is the same as the baseline model [105]. If the estimated coefficients of the three interactions are significantly positive, fiscal decentralization inhibits enterprise green development by generating resource mismatch. Table 6 reports the estimation results of the moderating effect of low-carbon governance on enterprise green development by fiscal decentralization and resource mismatch. Comparing the data in columns (1)-(6) of Table 9, we can get the following results: ① In Table 9 (4), the coefficient of the three-way interaction of fiscal decentralization, low-carbon governance and capital mismatch index (FIN×FREQU×TK) is negative (-0.016) and not significant, indicating that fiscal decentralization does not affect enterprise green development through capital market mismatch; ② In Table 9 (6), the coefficient of the three-way interaction of fiscal decentralization, low-carbon governance and labor mismatch index (FIN×FREQU×TL) is significantly positive (0.553). At the same time, the coefficients of low-carbon governance and labor mismatch index are both significantly negative, indicating that fiscal decentralization inhibits local enterprise green development by causing labor market mismatch. Therefore, this result verifies hypothesis 4.

**Table 9:** The impact of fiscal decentralization on resource misallocation.

	(1)	(2)	(3)	(4)	(5)	(6)
VARIABLES	GRE	Carbon_dioxide	GRE	Carbon_dioxide	GRE	Carbon_dioxide
FIN×FREQU		1.933***				
		(0.064)				
FIN×FREQU×TK				-0.016		
				(0.071)		
FIN×FREQU×TL						0.553***
						(0.039)
Carbon_dioxide	0.135***		-0.151***		0.079***	
	(0.013)		(0.020)		(0.012)	
FIN		-0.004***		-0.001***		-0.000***
		(0.000)		(0.000)		(0.000)
TK				0.008***		
				(0.001)		
FREQU		-10.551***		-1.258***		-2.014***
		(0.350)		(0.147)		(0.154)
TL						-0.016***
						(0.001)
Constant	0.019***	0.066***	0.022***	0.058***	0.020***	0.046***
	(0.002)	(0.005)	(0.002)	(0.005)	(0.002)	(0.005)
Observations	256,555	256,555	256,555	256,555	256,555	256,555
R-squared	0.753	0.150	0.708	0.147	0.759	0.150
Firm FE	YES	YES	YES	YES	YES	YES
Province FE	YES	YES	YES	YES	YES	YES
Year FE	YES	YES	YES	YES	YES	YES

Note: FIN×FREQU represents the intersection of fiscal decentralization and low-carbon governance. FIN×FREQU×TK represents the intersection of fiscal decentralization, low-carbon governance, and capital misallocation. FIN×FREQU×TL represents the intersection of fiscal decentralization,0 low-carbon governance, and labor misallocation. Firm FE of YES means that this part of the study controls for firm fixed effects. Province FE of YES means that this part of the study controls for province-fixed effects. Year FE of YES means that this part of the study controls for time-related fixed effects.

*** represents significance levels of 1%.

## 7. Conclusion and policy implications

As a tool of environmental regulation, whether low-carbon governance can promote the green transformation of enterprises is an urgent practical problem that needs to be solved. Therefore, this paper uses the 2SLS model based on instrumental variables and the mechanism test model to discuss the impact of low-carbon governance on the green development of enterprises and its mechanisms. Combined with the decentralization characteristics of China’s low-carbon governance, this paper analyzes the regulatory effects of fiscal decentralization on the impact of low-carbon governance on the green development of enterprises. The main conclusions are as follows.

First, low-carbon governance has significantly promoted the green development of enterprises and passed a series of robustness tests. Low-carbon governance will impose severe penalties on enterprises that exceed carbon emission standards, such as shutting down, and raise the cost of enterprise pollution and encourage enterprises to carry out green transformation. Scholars have identified the internal factors that promote the green development of enterprises, such as social responsibility, corporate governance, technological innovation, corporate governance, and green finance policy. This paper shows that low-carbon governance is also a feasible way to promote the green development of enterprises [17–21].

Second, the mechanism test shows that industrial structure upgrading, and technological innovation are the two main channels for low-carbon governance to drive the green development of enterprises. This article, therefore, contributes to this literature. By deeply exploring the impact of industrial structure upgrading and technological innovation, this paper allows for a more comprehensive understanding of the internal mechanism of how low-carbon governance affects the green development of enterprises.

Third, heterogeneity analysis shows that low-carbon governance can significantly promote the reduction of carbon dioxide emissions of state-owned enterprises and private enterprises. In contrast, private enterprises have better green development effects. These findings are consistent with existing literature showing that the impact of low carbon governance on total factor productivity was consistent with that of green total factor productivity [12, 106]. Moreover, the effect of low-carbon governance is better in the eastern region and labor-intensive enterprises.

Fourth, fiscal decentralization reflects the fiscal power distribution relationship between higher and lower governments. It plays a regulating role in the effect of low-carbon governance to drive the green development of enterprises. Fiscal decentralization mainly causes the misallocation of labor market resources rather than capital resources, thus weakening the effect of low-carbon governance on the green development of enterprises.

The research findings of this study hold significant policy implications and can serve as a reference for environmental governance and green development transitions in developing countries. Firstly, low-carbon governance policies should prioritize green development as their objective and actively guide industrial structural upgrades and technological innovations, harnessing the "structural effect" and "technological effect" of low-carbon governance. This entails promoting the transformation of industries from low value-added to high value-added, shifting from high energy consumption and high pollution to low energy consumption and low pollution, and transitioning from extensive to intensive modes of operation. Enterprises should undergo comprehensive transformations in technology, market strategies, and management practices. Secondly, the government needs to implement differentiated low-carbon governance measures tailored to different ownership types of enterprises. Given the heterogeneity in the green development effects of low-carbon governance across various ownership types, a targeted approach should be adopted to implement policies that optimize governance structures flexibly and drive green development effectively. Lastly, it is crucial to optimize the setting of fiscal decentralization assessment indicators and harness their positive role in low-carbon governance. A lower degree of fiscal decentralization and reduced mismatch in labor resources have been shown to promote enterprise green development. However, in the reform process, it is essential to optimize fiscal decentralization to mitigate its adverse effects on low-carbon governance when executed by local governments. Addressing issues arising from fiscal decentralization-induced labor resource misallocation requires appropriate measures to reduce its inhibitory impact on the effectiveness of low-carbon governance. In conclusion, these policy implications provide valuable insights for developing countries seeking to enhance their environmental governance and facilitate the transition toward sustainable green development.

Although this paper has identified the impact of low-carbon governance on enterprise green development, analyzed and discussed it from the perspective of fiscal decentralization, and given corresponding policy suggestions, it still has some limitations. First, the synergistic mechanism between low-carbon governance and other environmental regulations is not identified. Second, although this paper has studied the impact of China’s low-carbon governance on enterprise green development, it lacks a comparative study with other countries’ low-carbon governance. In general, both low-carbon governance and enterprise green development are long-term processes. Therefore, future research could start from the above aspects and further explore the long-term mechanism of low-carbon governance to promote enterprise green development.
